# Development and analysis of quality assessment tools for different types of patient information – websites, decision aids, question prompt lists, and videos

**DOI:** 10.1186/s12911-023-02204-5

**Published:** 2023-06-21

**Authors:** Lena Josfeld, Jutta Huebner, On behalf of the Working Group Prevention and Integrative Oncology in the German Cancer Society

**Affiliations:** Department of Hematology and Medical Oncology, University Hospital Jena, Jena, Germany

**Keywords:** Patient information material, Quality assessment tool, Evaluation of development process, Inter-rater concordance, Oncology

## Abstract

**Objective:**

Our working group has developed a set of quality assessment tools for different types of patient information material. In this paper we review and evaluate these tools and their development process over the past eight years.

**Methods:**

We compared the content and structure of quality assessment tools for websites, patient decision aids (PDAs), question prompt lists (QPLs), and videos. Using data from their various applications, we calculated inter-rater concordance using Kendall’s W.

**Results:**

The assessment tools differ in content, structure and length, but many core aspects remained throughout the development over time. We found a relatively large variance regarding the amount of quality aspects combined into one item, which may influence the weighting of those aspects in the final scores of evaluated material. Inter-rater concordance was good in almost all applications of the tool. Subgroups of similar expertise showed higher concordance rates than the overall agreement.

**Conclusion:**

All four assessment tools are ready to be used by people of different expertise. However, varying expertise may lead to some differences in the resulting assessments when using the tools. The lay and patient perspective needs to be further explored and taken into close consideration when refining the instruments.

**Supplementary Information:**

The online version contains supplementary material available at 10.1186/s12911-023-02204-5.

## Introduction

Many patients in general and cancer patients in particular show a high need for information regarding their condition and treatment options [1–3]. With decisions of an often large scope and of far-reaching consequences, information and knowledge provide an essential basis for patients’ active involvement in the decision-making process [2, 4, 5]. While a personal conversation with the treating physician tends to be the preferred source of information [4, 5], evidence also suggests that patients may feel underinformed [6, 7] after consultations.

Consequently, many patients (as well as caregivers) resort to autonomous searches for additional information for which the internet has become a major source [5, 8–11]. One of the main challenges of potentially vital importance when searching for online information is to determine the quality and reliability of the search results [12], especially for people who have little experience with the subject itself or with the pitfalls of the internet in general. Most patients searching for information online are lay people regarding medical matters; when it comes to cancer patients, their average age is relatively high and, consequently, their average digital literacy often relatively low.

Clinicians, on the other hand, are often confronted with the problem of limited time opposing their patients’ need for in-depths information. Good-quality material to refer patients to is one possible way out of this dilemma. But again, it can be quite a challenge to determine how reliable the material is or how appropriate and understandable for the target audience.

The necessity for support in finding and assessing good-quality information online is evident; different sets of criteria exist in this regard, but they vary and are not commonly known to the average user. Authors and publishers of (online) health information only partly adhere to them [13–18], which may be due to a lack of awareness or to the criteria being too difficult to handle especially with regard to different types of patient information.

Websites in general provide a wide variety of types and quality of information. Authors range from established health care providers, individual practitioners, self-help groups, pharmaceutical companies to personal blogs and more. As a sub-genre of online information, videos provide a particular format which can be especially helpful for and is often preferred by people with literacy problems or simply those preferring audio-visual information to reading text on websites.

A more specific format designed to assist patients are patient decision aids (PDAs). They aim to provide the required information, which allows patients to assume an active part in the decision-making process regarding treatment, examinations or screenings.

Another tool for supporting patients’ information gathering are question prompt lists (QPLs). They are designed to aid communication between patients and physicians during consultation so that both sides receive the important information required for informed decisions and optimal treatment.

For all information formats, quality assessment to distinguish high-quality material from low-quality or even false and dangerous material is important. For written material there is a national and international consensus on quality criteria as specified in publications and instruments like afgis, DISCERN or HONcode [19–23]. These emphasise first and foremost the importance of up-to-date evidence-based information from transparent and unbiased sources which can be understood by lay people. For other formats, no such criteria and instruments exist, which consider the specialities of the respective format.

The aim of our working group therefore was, to develop a set of instruments for modern formats based on the consented criteria. Over the course of the past eight years (2014—2022), we have designed quality assessment tools for different types of (online available) patient information: websites [13–16], patient decision aids (PDAs) [article submitted to BMC Medical Informatics and Decision-Making], question prompt lists (QPLs) [17], and videos.

The aim of this study is to analyse the resulting instruments with respect to applicability and usefulness as well as inter-rater concordance. From there, we try to create a template of core criteria plus additional ones that can be used to evaluate the quality of different types of patient information.

## Material and methods

### Development and structure of the tools

To develop the respective instrument, we used a merged set of criteria developed in an expert consensus process from several renowned instruments, guidelines and assessment tools on written patient information. With the internet having become an increasingly important source of information, we started out with the development of criteria to assess the quality of websites containing patient information [13]. Based on those, the criteria for (online available) QPLs [17] and PDAs were developed next [article submitted to BMC Medical Informatics and Decision-Making]. The most recent tool eventually focussed on videos [article in progress].

For every format, we first systematically searched existing literature for instruments and criteria assessing the quality of patient information in the respective format, as well as general quality criteria for patient information. Items were then selected and adapted where necessary to fit the context – websites, PDAs, QPLs or videos. The resulting item lists were checked for completeness by at least three researchers, and context-specific items were added where necessary. For each format, a preliminary tool was developed and tested on two or three examples of the respective format by two to four different raters. Based on the results, the item lists and instructions were refined. After a second test run, the tools were finalized and then tested on a larger number of examples. Detailed information on the development process of each tool and on the incorporated sets of previously existing criteria can be retrieved from the respective publications.

Each resulting tool comprises a list of single items which can be rated on a three-point Likert scale: completely fulfilled, in part fulfilled, not fulfilled. The tools differ in the assigned values: some use values between 0 and 2, others between 1 and 3. The items are sorted in two categories: content criteria and formal criteria. The assessment tool for videos contains “user-oriented criteria” as a third category.

The tools differ in their methods of calculating the final results of the assessment. The original tool for websites only builds sums of the assigned values of each item. The tools for PDAs and QPLs add the option to use the mean values of each subcategory of items, as well as transforming the final result of an assessment into percentages. This last step allows for comparability if certain categories are not applicable to some of the publications which would otherwise leave those publications with a disadvantage in overall points.

### Material

During the time from 2011 to 2022, these four assessment tools were applied to different information material. For each topic, we simulated a patient internet search and derived the top hits which then were assessed using the tool. Details of the search strategies and the information finally assessed are described in the respective publications.

- Websites:
  - o 77 websites containing patient information on oncology, evaluated by two raters (one medical student, one physician) [13]
  - o 60 websites on cancer diets, evaluated by two raters (two students of medicine and medical informatics) [14]
  - o 50 websites on thyroid cancer, evaluated by four raters (one patient, two other lay people, one physician) [15]
  - o 96 German websites of oncologists and non-medical practitioners, evaluated by two raters (both medical students) [16]
  - o 40 websites containing information on diet and nutrition for cancer patients, evaluated by four raters (two medical students, two lay people) [article submitted to Archives of Public Health]
- PDAs: 22 PDAs evaluated by four raters (two medical students, two physicians) [article submitted to BMC Medical Informatics and Decision-Making]
- QPLs: 46 QPLs specifically for cancer patients. evaluated by four raters (two medical students, two physicians) [17]
- Videos:
  - o 26 videos on complementary and alternative medicine (CAM) in oncology evaluated by four raters (two medical students, two physicians) [article in progress]
  - o 30 videos on diet and nutrition for cancer patients evaluated by four raters (two medical students, two physicians) [article in progress]

### Statistics

In order to assess the agreement between different raters of the same material Kendall’s coefficient of concordance (Kendall’s W) was calculated, it being a relatively powerful alternative to the often used Cohen’s kappa, which is appropriate for nominal data but not for the ordinal data of these assessments [24]. Within each study, publications were ranked for each rater separately according to their final value. Kendall’s W was then calculated for each type of publication using IBM SPSS Statistics Version 27.0. Level of significance was set at *p* < 0.05. We calculated overall coefficients including all raters, as well as coefficients for subgroups where possible, differentiating between expert raters (physicians), semi-experts (medical students) and lay raters.

There is no universal interpretation of the resulting values of W, since interpretation depends on the field of application. In accordance with current literature and taking into account the complexity of most of the evaluated material, we classify concordance levels over 0.5 as acceptable, over 0.65 as reasonable, over 0.8 as strong, over 0.9 as very strong [25, 26].

## Results

### Comparison of the tools

Overall, the four assessment tools contained a lot of similarities with regard to the collected quality criteria. A detailed comparison of the tools is presented in Table 1. The website tool serves as basis for comparison since it was the first to be developed and the development of subsequent tools used it as a basis to varying degrees.

**Table 1:** Comparison of the four quality assessment tools based on the original tool for websites

Websites	PDAs	QPLs	Videos
*Content criteria*			
Expertise	Item split in two, referring authors ‘ qualifications and inclusion of patients’ perspective; both assigned to formal criteria	Item refers to experts and potential users (physicians and patients) – assigned to formal criteria	No explicit item included
Objectives (Are they evident? Are they being met? Is the target group specified?)	Item referring only target group	Item carried over with small changes	Item split in two, referring objectives and target group – the latter is assigned to user-oriented criteria
Is the writing balanced and unbiased?	Item adapted referring pros and cons of treatment/screening methods	Item carried over	No explicit item included
Precision (Are statements and information precise?)	No explicit item included	Item carried over	Item carried over with added aspect of numerical data/figures being given
Relevance (Are information and illustrations relevant for users? Is the context of presented data relevant for users/target group?)	No explicit item included	Item carried over	Item carried over and assigned to user-oriented criteria
Complementarity (Does the publication support and facilitate shared decision-making?)	Item changed: different ways of reaching a decision are made explicit	Item changed: objective to support, not to replace a consultation	Item changed: no replacement for consultation
Standards of information (scientific evidence, up to date)	Item assigned to formal criteria; specified to communicating the quality of scientific evidence, including a lack of evidence	No explicit item included [not applicable to QPLs]	Item specified to evidence-based information
No statements regarding topics without ascertained information (Does the publication comment on areas for which no ascertained information is available?)	No explicit item included	No explicit item included [not applicable to QPLs]	No explicit item included
Detailed information about treatment procedures (Does the publication describe the benefits of each treatment procedure, the mode of action of each treatment procedure, the risks of each treatment procedure, possible consequences of non-treatment, how the treatment procedures affect quality of life?)	Item split in two	No explicit item included [not applicable to QPLs]	Item carried over
Does the publication contain details of supplementary aids and information?	No explicit item included	Two items, referring additional information and the indication, that there are sources of information beyond the consultation with the physician	No explicit item included
Statements are based on patient-relevant endpoints: Improvement in health status, reduction in the duration of the disease, prolongation of life, reduction of side effects	Similar item included in formal criteria (evidence from studies populations which are similar to the target group)	No explicit item included	No explicit item included
Are the illustrations appropriate/understandable? (Additions of appropriate graphical representations are useful)	Item specified: illustrations support content without distracting	Item specified: illustrations, if used, support information processing	Item specified: illustrations adequately support verbal information
Consideration of layout aspects	Item extended: layout aspects support the content and present positive and negative aspects of all options in a neutral manner	Item split in two: the formatting (font, font size) is easy to read + form of presentation does not grade the different items/topics	No explicit item included
Communication of risks (More than a general, verbal presentation of risks. Presentation of potential loss and gain side by side)	Item assigned to formal criteria; specified regarding target population and consistent scale	No explicit item included [not applicable to QPLs]	No explicit item included
Quality assurance procedures	Item assigned to formal criteria; specified to include professionals and lay users	Item assigned to formal criteria; specified to include professionals and lay users	Item assigned to formal criteria
Clear arrangement of information (Is the information clearly presented? Is there a search function?)	Item carried over	Item split in two: clear structure + comprehensible order of items	Item split in two: clear structure + order of information facilitates understanding
Completeness of information	Item separated into all relevant categories	Item carried over (all relevant topics listed in item description)	Item carried over
Lack of evidence is openly communicated	Item assigned to formal criteria	No explicit item included [not applicable to QPLs]	Item carried over
		A user guide is included	
		No repetition/redundancy among items	
*Formal criteria*			
Use of language that supports participation and is adapted to the target group	Item split in two and assigned to content criteria: language understandable for target group + guiding through the decision process to reach an individual decision	Item split in three, assigned to content criteria: language understandable for target group + encouragement to discuss option with physician + guiding through the conversation to attain all relevant information	Item assigned to user-oriented criteria
Transparency (Are authors and data sources of the information named? Information about the provider? Disclosure of funding/sponsors? Disclosure of advertising policy? Separation of advertising and editorial content?)	Item split in three: developer information + funding + potential gains and losses of developers	Item split in four: developer information + funding + potential gains and losses of developers + clear separation of content and advertising	Item carried over
Data protection (Is there information on data security? How is personal data used and protected)	Item changed: security for personal information entered into the website	Item split in two: explicit information on data use and protection + security for personal information entered into the website	No explicit item included
Information on sources (Is there a clear indication of the sources of information used to produce the publication (in addition to the author or producer)? Is it clearly stated when the source material was produced?)	Item split and extended to four: scientific evidence cited + description of evidence quality (incl. lack of evidence) + additional information on selection process of cited evidence + last update of PDA given	Item changed: indications on up-to-dateness + steps for selecting question by creators are explained	Item split in two
Scientific knowledge about the presentation of numbers and outcomes is taken into account	Item split in two and specified: probabilities correctly described as uncertain and put into context with other events + description of how probabilities have been calculated	No explicit item included	Item carried over and assigned to content criteria
User involvement (opportunity for feedback from users; patients are involved in the process of information production)	No explicit item included	Item carried over	Item carried over and assigned to user-oriented criteria
	Low-threshold access for all patients within the target group without additional costs	Low-threshold access for all patients within the target group without additional costs	
	Offers options other than reading (e.g. audio, video or in-person discussion)	Offers options other than reading (e.g. audio, video or in-person discussion)	
	*for web-based material*		
	Good navigation options integrated into the website	Good navigation options integrated into the website	
	Easy to return to after linking to other websites	Easy to return to after linking to other websites	
	Offers search for keywords	Offers search for keywords	
	Can be printed as single document	Can be printed as single document	
	Offers feedback on personal health information where they can be included	Offers feedback on personal health information where they can be included	
		Easy to find online	
*Medium-specific criteria*			
	Helps to recognise that a personal decision has to be made	Points out that users can make their own selection of items/questions	Video description (Do title and content match? Does the description give a summary of the video content? Is the focus of the video recognisable?)
	Helps to know about the different options and asks patients to think about which positive and negative features of the options are most important to them	Points out that the formulations for the discussion with the doctor can be individually adapted and changed, and gives examples	Feasibility (Does the video clearly identify at least one action that users can perform? Does the video speak directly to users when describing the activity? Does the video break down each activity into manageable steps?)
	Explains that personal values can influence decisions		Audio quality (Background noise avoided? Supporting background music, signal tones or similar? Volume appropriate? Speed of speech appropriate? Speaker easy to understand?)
	Improves the match between the options finally chosen and the values and characteristics that are most important to the patient		Visual quality (Objects and people well lit? Image sharp? Camera movement appropriate? Text in video easy to read? Visual material easy to recognise?)
	Provides guidance in formulating values relevant to the decision		
	Describes both options of letting specific values impact the decision or not		
	Helps to implement each step in relation to the decision form chosen		
	Describes different aspects of options to help patients imagine what it is like to experience the physical, emotional and social impact of doing so		
*If other patients’ stories/testimonials are included:*	Stories describe both positive and negative experiences		
	Stories enable relating to steps others have made in their decision-making process		
	Stories introduce listed option and possible outcomes		
	Describes steps to select the stories and to verify contained information		
	Statement on informed consent included		
	Disclosure of financial or other reasons for patients to have shared their stories		

A number of aspects stood out when comparing all four tools:

- When assorting items into main groups, most tools differentiate between content criteria and formal criteria, but the tool for videos opened a third main category of user-oriented criteria.
- Several items seem to be difficult to distinctly assign to either content or formal criteria; their assigned category differed across tools.
- There were rather vast differences regarding the number of aspects combined into one item. While some tools contained fewer items comprising multiple questions, other tools separated those questions into multiple items. As a result, certain aspects may be weighted differently between tools regarding their impact on the final score and thus regarding their importance for a final judgement of good or bad quality.
- The majority of items can be applied to different types of material. The tool for PDAs contained the largest number of specific items only applicable to this type of material; a few specific items were created for QPLs and videos as well.

Looking at the changes over time, the following key evolvements were noticeable:

- Several items were altered to focus more on whether the material catered appropriately to its specific target group.
- The original idea that patient information should not touch on areas without confirmed information was dropped to focus on the demand for open communication of missing evidence.
- Additional criteria for web-based material were added while developing the tools for PDAs and QPLs, which would be equally applicable to and important for websites.

On the other hand, some of the original items from the website assessment tool were dropped in one or several of the later tools, even though they would have been applicable – like expertise of the authors/creators (dropped for videos), relevance of the information (dropped for PDAs), details on supplementary aids and additional information sources (dropped for PDAs and videos), focus on patient-relevant endpoints (dropped for QPLs and videos).

The apparent length of the tools differs considerably, which is due to a large extent to the different ways in which quality aspects were combined or separated into individual items, as can be seen in Table 2. Medium-specific items also contributed to this effect. A dissection of all four tools into individual quality aspects that are considered within the items is provided in additional file 1 (original German versions).

**Table 2:** Comparison of length

	*Websites*	*PDAs*	*QPLs*	*Videos*
*Number of items*	24	42	42	19
*Total number of aspects considered*	47	57	58	52

### Inter-rater concordance

All tests of overall agreement between the raters were significant on a 5% level, meaning that the null hypothesis W = 0 (no agreement among raters) could be rejected with 95% certainty in all applications of the tool. When differentiating for subgroups among raters (physicians, medical students, lay people), one tests did not reach significance on a 5% level, only if allowing for a risk of error of 10%: agreement between physicians on QPLs (*p* = 0.097). It remains unclear whether the differences of the two ratings originated in factors pertaining to the tool, the source material, or the raters themselves.

Table 3 gives an overview of the different levels of concordance between the raters, calculated for subgroups of raters where possible.

**Table 3:** Inter-rater concordance of all past applications of the four assessment tools

Source material / concordance^a^	Overall	Physicians	Medical students	Lay people	Other
Oncology websites—total scores (n = 77; 2 raters)	.881	/	/	/	
*Oncology websites—content scores*	.867	/	/	/	
*Oncology websites—formal scores*	.794	/	/	/	
Websites on cancer diets (n = 60; 2 raters)	.944				
Thyroid cancer websites (n = 50; 4 raters)	.516	/	/	.739	.725 (patient—physician)
Oncologists’ and NMPs’ websites (n = 96; 2 raters)	.761	/	/	/	
*Oncologists’ websites (n* = *49)*	.811	/	/	/	
*NMPs’ websites (n* = *47)*	.691	/	/	/	
Websites on diet and nutrition (n = 38; 4 raters)	.461	/	.673	.755	
*Websites on diet and nutrition—content scores*	.622	/	.795	.781	
*Websites on diet and nutrition—formal scores*	.525^c^	/	.703^c^	.620^c^	
*Websites on diet and nutrition – DGE*^*b*^* criteria*	.578^c^	/	.653^c^	.659^c^	
Oncological PDAs (*n* = 22; 4 raters)	.663	.607	.909	/	
Oncological QPLs (*n* = 46; 4 raters)	.710	.640 (*p* = .097)	.910	/	
Videos on complementary and alternative medicine (*n* = 24; 4 raters)	.851	.939	.910	/	
*Videos on complementary medicine (n* = *13)*	.752	.928	.877	/	
*Videos on alternative medicine (n* = *11)*	.899	.961	.930	/	
Videos on diet and nutrition (*n* = 29; 4 raters)	.842	.868	.899	/	

^a^Kendall’s coefficient of concordance (Kendall’s W): 0 = no concordance, 1 = perfect concordance; all tests significant at *p* < 0.05 with two exceptions given in brackets (*p* < 0.1)

^b^German Nutrition Society (Deutsche Gesellschaft für Ernährung)

^c^Very little variance in scores resulted in many identical/shared ranks, making the interpretation of Kendall’s W difficult

In 7 out of 9 applications of the tool, all inter-rater concordances were of 0.6 or higher. The lowest concordance resulted from the assessment of websites on diet and nutrition for cancer patients (Kendall’s W over all 4 raters: 0.461 (total scores), 0.622 (content scores), 0.525 (formal scores), 0.578 (specific criteria of the German Nutrition Society—DGE)), where a high number of shared ranks complicates Kendall’s W as a measure for concordance.

The ratings of websites on thyroid cancer differed particularly between subgroups of raters: concordance between lay people was good at W = 0.739, while the other two raters consisting of one physician and one patient showed similarly good concordance at W = 0.725.

A general comparison of overall concordance rates to those within subgroups of raters showed that concordance within subgroups is almost always higher than the overall concordance. The exception occurred in the assessments of PDAs and QPLs, where concordance among physicians was lower than the overall concordance (physicians and medical students).

## Discussion

The assessment tools for patient information which were regarded in this article, show some potential for their application not only within scientific research but also in clinical practice, possibly more so for medically trained than for lay people.

We found an overall good inter-rater concordance when tools were applied by medical students, physicians and lay people. Lowest concordance among all raters occurred for websites on thyroid cancer, websites on diet and nutrition, and oncological PDAs. For websites, these low levels are at least partly founded in differences between subgroups, as the concordance within those is substantially higher.

A pattern evolved of concordance within subgroups being usually better than the overall concordance – with the exception of concordance among physicians who evaluated PDAs and QPLs, which was relatively low (while that among medical students was very high). Each pair of physician raters was different, so the results cannot be attributed to differences in opinion of two particular people. This phenomenon needs to be studied in more detail: The physicians’ different views of the tools or the source material may provide valuable insight on how to assess the quality of patient information.

The only patient acting as rater in these studies had a higher concordance with the physician than with the other two lay raters. This result can easily be explained if one assumes that this particular patient had already gained considerable experience and knowledge about their disease. However, this is an isolated incident. At which point patients’ expertise resembles more that of a physician than that of lay people unaffected by a specific disease would need to be a subject of further research.

Another interesting result was the substantially lower concordance among the same raters (two medical students) when evaluating non-medical practitioners’ websites as opposed to oncologists’ websites. A possible explanation may be that the wide variety of topics listed and methods offered on these websites requires rather extensive knowledge in order to assess the quality of the information – a type of knowledge that is not typically taught in medical school, so the expertise of the raters on these particular subjects may have differed considerably in this regard.

Again, this is an isolated incident which calls for more in-depth research. Considering all of the described aspects, however, the question clearly arises how well people with less expertise on medical topics – which applies to the vast majority of patients – will be able use assessment tools like these or other previously published lists of criteria to adequately assess the quality of patient information. Especially PDAs may be difficult to evaluate even for medical experts, as their objective is not only to inform patients of medical topics but also to assist in an often difficult process of reaching a decision about their health. Evaluating their quality may require expertise not only in the particular medical field but also in the many intrapsychic and interpersonal processes involved in human decision-making.

Looking at the actual content of the tools, it may not be advisable to try and sort items into two large categories of content and formal criteria, since many aspects appear to touch on both areas. However, grouping items into smaller categories allows to control the weighting of certain aspects by the number of items within each category. Final values can thus either be calculated on the basis of individual items (categories with more items have a higher impact on the overall result) or on the basis of each category’s mean value (every category contributes equally to the overall result).

Other sets of criteria in international literature also tend to use main categories [20, 27–29]. Their number, contents and degree of detail, however, differ considerably. The difficulty of assigning items to clearly defined, selective categories appears to be a universal problem.

The differences between the tools in this review revealed some interesting developments over time. Some can be understood as a shift in focus which followed a certain ideal. For example, not dismissing topics without solid evidence allows the information material to educate about controversial or even potentially harmful topics which lack scientific evidence but may be of interest to patients. Leaving these topics out could lead to patients looking elsewhere for information and likely finding them in more problematic contexts. Including them may therefore even be beneficial; the quality criterion here is the clear communication of lack of evidence and what this means in terms of, for example, effectiveness and safety of a treatment.

Other changes were items added to the assessment tools for PDAs or QPLs which are applicable to websites as well and should be integrated into the website assessment tool for future use.

Some items of the original tool appear to have been dropped or shortened in order to streamline the tool or because these items would be difficult to assess or understand, especially for laypersons (e.g. whether field tests were carried out to evaluate the effectiveness of a PDA or QPL). Other items seem to have disappeared without externally apparent explanation.

Overall, some criteria seem to be essential when assessing information in order to ensure that no false information is provided and that patients are able to correctly understand and apply the information to their individual situation. Other criteria are of a more supplementary nature and add value to the material (such as providing further resources, feedback options for users etc.).

A final difficulty arises when applying one of the tools presented here to a single publication: No thresholds have yet been defined to differentiate between, for example, low, acceptable, and good quality. So far, transforming the final value into percentages offers the best option for an individual judgement on how well the assessed material meets the expectations. Again, this problem occurs with most other evaluation tools as well.

A 2005 review by Bernstam et al. [30] had already found a plethora of different evaluation criteria and tools but judged most of them unfit for practical use by patients or even by physicians. Since then, some lists of criteria have become more established, especially in research but also as recommendations for patients. But the usability of such tools for the latter tends to come at the cost of comprehensiveness. Restricting a tools length to make it more approachable will mean reducing the quality aspects it can cover or combining many of them into a few items, which risks reducing the discriminatory power and informative value. A possible solution to this dilemma may be the use of core criteria and supplementary once – the former constituting a strictly necessary set of criteria that determines whether a publication is trustworthy, the latter giving additional information about its value to users.

Research is needed regarding such tools’ sensitivity to a rater’s expertise. An in-depth analysis may reveal which quality aspects can be accurately evaluated independently of expertise, which items require detailed explanations or need to be broken down into several items in order to enhance inter-rater concordance across different levels of expertise.

### Limitations

The result presented in this review are limited in their conclusiveness for a number of reasons. Methodically, the small number of raters in the original studies limits the conclusiveness of concordance rates. The differences in number and background (regarding expertise) of the raters in each study potentially reduce their comparability. Kendall’s coefficient of inter-rater concordance is based on ranks which ultimately results in a comparison of intra-individual benchmarks of the raters. The tools appear well reliable when used to compare several publications of patient information and ranking them. However, their reliability regarding the absolute value of an assessment’s result still needs to be confirmed, which would be the basis for an objective measure of the kind of a threshold above which a publication can be considered good quality.

Another difficult aspect are the limited applications by lay-persons. This was due mainly to the difficulty in recruiting oncological patients who are already pressed for time and energy, to partake in these often lengthy evaluations. On the other hand, researchers need additional resources when presenting lay people with such a vast amount of information as has been used in these studies, to afterwards explain the quality of the presented material and especially to point out any false information that may have been included, in case the assessment tools did not work sufficiently well for the test subjects. So far, raters of varying expertise but mostly from within the field of medicine in general and oncology in particular have used the tools. The results show potential but in order to evaluate the applicability of these tools in clinical practice, an effort needs to be made to recruit lay raters and patients as well as physicians without active involvement in scientific research, which we hope to achieve in future studies.

In this context, the raters’ original expertise on the topic should also be assessed, as well as their expertise at the end of the assessment process in order to identify potential learning effects which may influence the results.

While the current tools may be used to assess individual content without comparison by using the results in percentages of an expected optimum, another future approach should be to find an evidence-based consensus on what is considered low, acceptable, or good quality on these scales to be clearly specified with their instructions for use.

### Conclusion

The assessment tools referred to in this article are ready to be used in practice, especially when comparing the quality of several publications of the same type. Researchers as well as clinicians and patients can employ them in their search for good-quality information material or to assess the quality of a given body of publications addressing patients.

They still leave room for improvement, though, regarding the optimal selection of criteria which would cover all relevant aspects without making the entire tool too complex and unwieldy. A suggestion for simplifying their use would be to find ways for integrating them into digital solutions.

The lay perspective is needed in order to assess how well these tools can really be applied in practice. To this end, a more extensive evaluation should be conducted which also assesses the raters’ original expertise on the subject of the information material. It is entirely possible, that especially for complex topics no assessment tool is sufficient to find good-quality information without first developing the user’s own expertise.

## Supplementary Information

**Additional file 1.** Comparison of all aspects within the tools’ criteria.

## Data Availability

The datasets analyzed during the current study is available from the corresponding author on reasonable request.

## Abbreviations

CAM: Complementary and alternative medicine
DGE: Deutsche gesellschaft für Ernährung [German Nutrition Society]
PDA: Patient decision aid
QPL: Question prompt list
